# Supplementary material for: High-resolution mapping of Ryd4Hb, a major resistance gene to Barley yellow dwarf virus from Hordeum bulbosum
Source: Theor Appl Genet. 2024 Feb 27;137(3):60. doi: 10.1007/s00122-024-04542-y (PMC10896957; doi:10.1007/s00122-024-04542-y)
Supplement: Supplementary file 1 — Supplementary file1 (PDF 72 kb) [file 122_2024_4542_MOESM1_ESM.pdf]

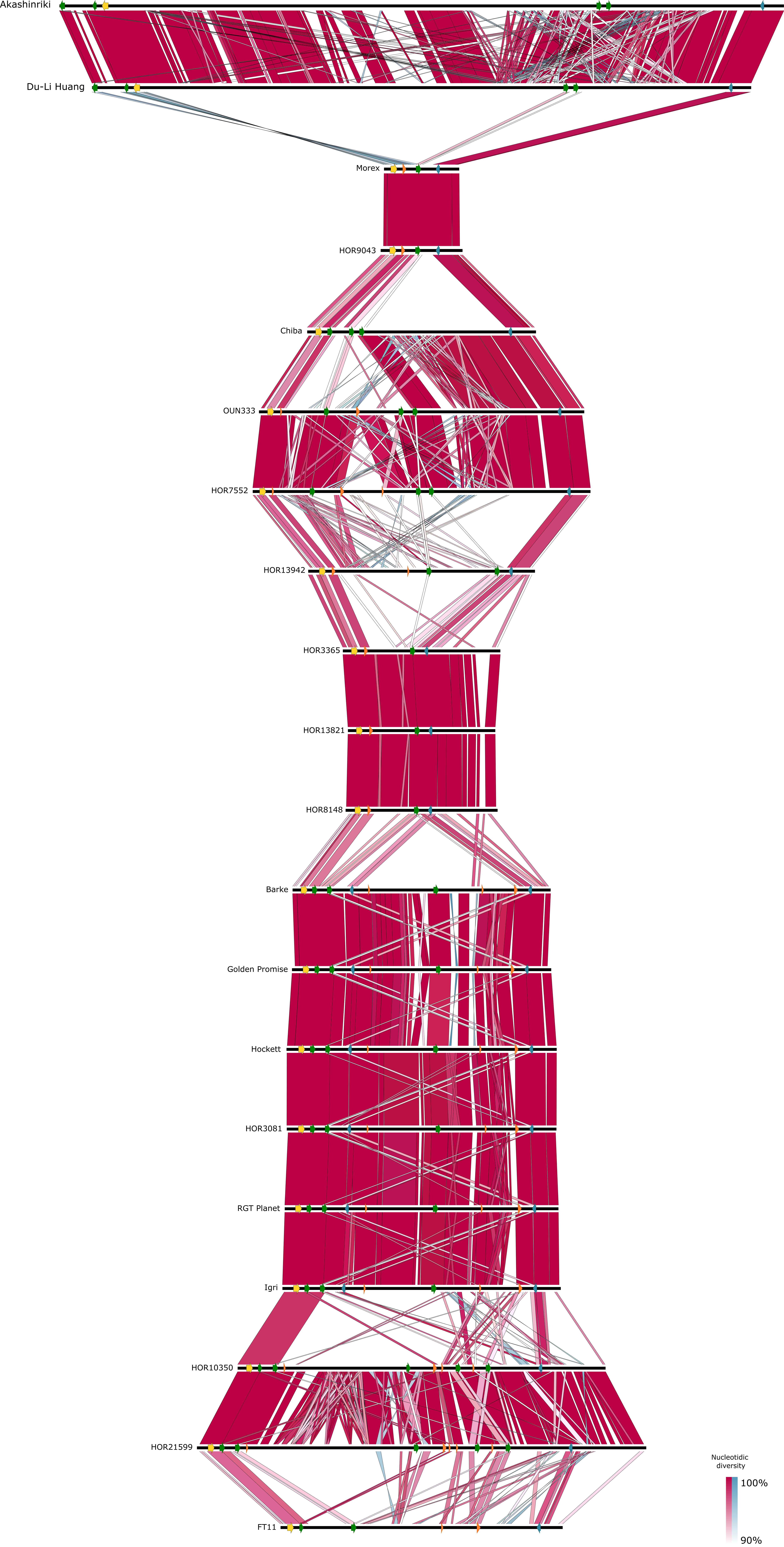

Supplementary Figure 1: Comparison of *Ryd4^{Hb}* in *H. vulgare* pangenome modified from Easyfig 2.2.5 output. Only fragments with more than 80% nucleotide identity are shown.
